# Intrathecal Delivery of Macromolecules to the Spinal Cord Enabled by Laser-Activated Perfluorocarbon Nanodroplets

**DOI:** 10.1021/acsnanomed.5c00207

**Published:** 2026-04-29

**Authors:** Robert J. Nikolai, Anthony Donsante, Jason J. Lamanna, Melissa Cadena, Anamik Jhunjhunwala, David Qin, Elena V. Vassilieva, Arya T. Oak, Shreya Vavilala, Matthew Byrne, Nicholas M. Boulis, Stanislav Y. Emelianov

**Affiliations:** † The Wallace H. Coulter Department of Biomedical Engineering, Georgia Institute of Technology and Emory University School of Medicine, Atlanta, Georgia 30332, United States; ‡ Department of Neurosurgery, Emory University School of Medicine, Atlanta, Georgia 30332, United States; § School of Electrical and Computer Engineering, 1372 Georgia Institute of Technology, Atlanta, Georgia 30332, United States

**Keywords:** Nanodroplets, Perfluorocarbon, Intrathecal, Cavitation, Spinal Cord, Drug Delivery

## Abstract

The lack of safe, minimally invasive strategies for targeted drug delivery to the spinal cord remains a major barrier to treating neurodegenerative disease and spinal injury. Intrathecally administered macromolecules, including gene therapy vectors, rarely penetrate spinal tissue at therapeutically relevant concentrations due to cellular barriers at the cerebrospinal fluid–spinal cord interface. Here, we demonstrate the application of laser-activated perfluorocarbon nanodroplets (PFCnDs) for nonsurgical, spatially controlled delivery from the subarachnoid space into spinal cord parenchyma. Following intrathecal injection in rats, transdermal laser irradiation produced a 7-fold increase in photoacoustic signal within the spinal cord parenchyma compared to nonirradiated controls, confirming spatially selective intraparenchymal nanodroplet delivery. Codelivery of 500 kDa FITC-dextran demonstrated approximately 1 mm penetration into the dorsal horn at irradiated sites, while nonirradiated regions showed only superficial perivascular accumulation. Neither nanodroplets alone nor laser irradiation alone produced intraparenchymal delivery, indicating that vaporization-induced cavitation is necessary for penetration. The PFCnDs (∼300 nm diameter) were engineered with a lipid shell, perfluorohexane core, and near-infrared absorbing dye for transdermal activation at 1064 nm. These results demonstrate that laser-activated nanodroplets can deliver macromolecular cargo into the spinal cord via lumbar puncture and transdermal irradiation, without surgical exposure.

## Graphical abstract

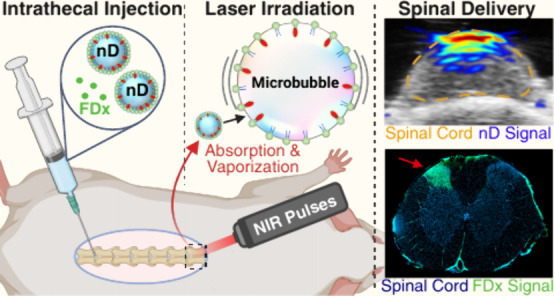

## Introduction

Spinal cord injury and neurodegenerative disorders are devastating clinical conditions with limited disease-modifying treatment options.^1^ Millions of patients worldwide live with progressive paralysis, severe sensory deficits, and chronic pain, yet emerging biologic therapies that could alter disease trajectories rarely reach their intended targets within the central nervous system (CNS). Systemically delivered therapies encounter poor permeability across the blood-spinal cord barrier^2^ and induce immune-related toxicity.^3^ Moreover, they lack spatial targeting within the spinal cord. Direct intraparenchymal injections are highly invasive, requiring a laminectomy for access and risking additional spinal trauma.^4^

Intrathecal administration delivers therapeutics directly into the cerebrospinal fluid (CSF), reducing systemic exposure and requiring lower dosages.^5,6^ Small-molecule and oligonucleotide therapeutics, including ziconotide and nusinersen, have achieved clinical success via this route.^5^ However, larger macromolecules such as viral vectors show limited spinal cord penetration.^7^ Molecular exchange between the CSF and spinal tissue occurs primarily through perivascular channels known as Virchow–Robin spaces.^8^ These channels and the entire spinal cord surface are lined by cellular barriers, including the glia limitans, that restrict macromolecular entry into the spinal cord parenchyma.^9–11^ For example, Householder et al. showed that a 100 nm nanoparticle population can distribute throughout the subarachnoid space in mice but does not meaningfully penetrate CNS parenchyma.^12^ Together, these challenges indicate the need for a new approach to safely induce localized permeabilization of the CSF–spinal interface for precise delivery of intrathecally administered therapeutics.

Focused ultrasound (FUS) and systemically administered microbubbles (MBs) are routinely used to open the blood-brain barrier (BBB).^13^ Unlike pharmacological permeabilization strategies, which produce diffuse, nonspecific opening and carry risks of neurotoxicity,^14,15^ FUS with microbubbles enables spatially targeted disruption. Building on this framework, we sought to apply cavitation-mediated barrier permeabilization to the CSF–spinal cord interface. However, the irregular geometry of vertebral bone and the narrow spinal canal distort FUS acoustic fields and promote standing waves that increase the risk of injury to adjacent neural tissue.^16^ Shimamura et al. demonstrated that insonated microbubbles can increase intrathecal spinal gene uptake in rats, but only after a laminectomy to avoid the acoustic challenges posed by vertebral bone.^17^ Moreover, microbubbles are too large (1–10 μm) to enter Virchow–Robin spaces, which typically measure fewer than 2 μm across.^8^ We therefore turned to a nanoscale agent.

Laser-activated perfluorocarbon nanodroplets (PFCnDs) are an alternative class of cavitation agents that can be selectively activated through optical absorption rather than acoustic focusing.^18^ Upon exposure to near-infrared (NIR), nanosecond laser pulses, these 300 nm droplets absorb the optical energy and undergo a rapid liquid-to-gas phase transition.^19^ This volumetric expansion generates localized mechanical forces that permeabilize nearby cellular barriers.^20,21^ While vertebral bone strongly scatters light, it exhibits minimal optical absorption at these wavelengths.^22^ Consequently, the laser energy diffuses through the spinal column without significant heating and is selectively absorbed by the PFCnDs. The optical energy required for nanodroplet activation in the spine can be delivered by laser pulses transmitted transdermally or potentially via an intrathecal catheter, thus enabling minimally invasive, targeted delivery. In addition, laser-activated PFCnDs have intrinsic photoacoustic contrast, enabling their utility as delivery reporters in the spinal cord.^23^

Here, we demonstrate that laser-activated PFCnDs enable nonsurgical, controlled, spatially-localized delivery of large macromolecules to the spinal cord. We designed and synthesized PFCnDs optimized for NIR activation and evaluated their in vivo performance in rats following intrathecal injection and transdermal laser irradiation. We quantified nanodroplet penetration into the spinal cord parenchyma of the lumbar enlargement using ultrasound and photoacoustic (US/PA) imaging. We confirmed intraparenchymal delivery of a ∼30 nm particle by confocal microscopy and demonstrated that delivery can be controlled by tunable parameters using fluorescent imaging. The overall purpose of this study is to present a new generalizable tool for delivering large-molecule therapeutics to the spinal cord.

## Methods and Materials

### Nanodroplet Synthesis and Purification

Nanodroplets were synthesized with a lipid shell composed of DSPE-PEG(2000) (18:0 PEG2000 PE, ammonium salt; Avanti Polar Lipids) and DSPC (18:0 PC; Avanti Polar Lipids), encapsulating a perfluorohexane core (FluoroMed, L.P.) and the near-infrared dye Epolight 3072 (Epolin, Inc.) with a reported peak absorbance at 1054 nm.

To prepare the lipid shell, 100 μL of a 25 mg/mL DSPE-PEG (2000) solution and 50 μL of a 10 mg/mL DSPC solution was added to a 10 mL pear-shaped flask. Here, 400 μL of Epolight 3072 solution (2 mg/mL in chloroform) was added to the same flask. The solvent was evaporated under reduced pressure using a rotary evaporator (Rotovapor, Büchi), yielding a dye-coated lipid film.

The dried film was rehydrated in 4 mL sterile phosphate-buffered saline (PBS), transferred to a 7 mL scintillation vial, and supplemented with 150 μL PFH. The mixture was vortexed for 10 s (Vortex Mixer, Fisher Scientific) and probe-sonicated (VWR, 180 W) in an ice-cold water bath for 1 min to generate nanodroplets.

The suspension was divided into two 2 mL centrifuge tubes and centrifuged twice at 300 × g for 3 min each (Mini-Spin, Eppendorf) to remove excess PFH and unincorporated dye; supernatants were transferred to fresh tubes and pellets discarded. To concentrate the formulation, the suspension was centrifuged again at 3000 × g for 3 min, the pellets were retained, and the supernatants discarded. Pellets were resuspended in 0.5 mL sterile PBS for final use.

### Nanodroplet Characterization

Nanodroplet size distribution was measured in PBS using a dynamic light scattering instrument (DLS, Zetasizer Nano ZS, Malvern Panalytical) at both room temperature (25 °C) and physiological temperature (37 °C). A spectrophotometer (Evolution 220, Thermo Scientific) was used to measure the optical absorption spectra of the dye-loaded nanodroplets. To minimize nonabsorptive extinction contributions, such as baseline scattering from the lipid shell, the normalized spectrum of unloaded nanodroplets was subtracted from the dye-loaded nanodroplet spectrum, yielding a corrected absorption profile for the complete formulation. Unloaded nanodroplets were synthesized using the same procedure described above, with the dye omitted. Nanodroplet concentration was measured using nanoparticle tracking analysis (NanoSight, Malvern Panalytical).

### Nanodroplet Cytotoxicity Assessment

Cytotoxicity was assessed in HEK293 cells. HEK293 cells were cultured in Dulbecco’s Modified Eagle Medium (DMEM, high glucose, Thermo Fisher Scientific) supplemented with 10% fetal bovine serum (FBS, Gibco) and 1% penicillin–streptomycin (Pen/Strep, Gibco) at 37 °C in a humidified incubator with 5% CO_2_. Cells were seeded in 96-well plates at an initial density of 25,000 cells per well and allowed to equilibrate for 24 h. Nanodroplets were diluted in culture medium from as-synthesized stocks (in PBS) to 1:100, 1:200, 1:400, 1:800, and 1:1600 (v/v), spanning the ∼1:200 working dilution commonly used for PA measurements; media-only wells served as controls. After 24-h exposure, wells were washed with PBS to remove any residual nanodroplets, and MTT solution (3-(4,5-dimethylthiazol-2-yl)-2,5-diphenyltetrazolium bromide) was added at 0.5 mg mL^–1^ in culture medium. Plates were incubated for 4 h, the solution was aspirated, and 200 μL of DMSO was added to each well for complete dissolution of the formazan salt. The absorbance at 590 nm was measured using a multiwell plate reader (Synergy HT, BioTek) to evaluate cell viability. Cell viability was normalized to untreated control wells.

### Animal Preparation and Intrathecal Injection

All animal experiments were performed following the protocols evaluated and approved by the Institutional Animal Care and Use Committee (IACUC) at the Georgia Institute of Technology (Ethics Approval Number. A100274). In each study, rats were randomly assigned to experimental groups.

Prior to intrathecal injections, female Sprague–Dawley rats of at least 90 days of age were anesthetized with isoflurane (Henry Schein) delivered in 100% O_2_ (Airgas) and were positioned prone in a stereotaxic frame. Dorsal fur was removed by shaving followed by depilatory cream to aid in transdermal optical transmission during laser irradiation. Proparacaine (0.5%, Henry Schein) was applied to the eyes to prevent dryness. Next, rats were placed prone with the forebody elevated ∼5 cm above the hindquarters to arch the spine and widen the interlaminar spaces. The L5–4 intervertebral space was identified by palpation, and a 5 mm midline skin incision was made using a No. 11 scalpel. A 1-in. Quincke spinal needle (BD, 405073) was advanced into the interspace, and correct placement into the intrathecal space was confirmed by observation of a tail or hindlimb flick. The needle stylet was then withdrawn to allow CSF to enter the hub. Using a micropipette, 30 μL of cerebrospinal fluid was withdrawn and immediately replaced with 30 μL of test solution, either sterile saline, nanodroplets alone, or nanodroplets mixed with 2.5% w/v FITC-dextran (MilliporeSigma), delivered directly into the spinal needle hub. A 1 mL syringe was preloaded with 30 μL of air and attached to the spinal needle. The solution was manually injected over the course of 30 s. The needle was held in place for an additional 60 s to allow the injection bolus to stabilize. Following slow removal of the needle, animals were positioned prone and flat for 20 min prior to either recovery or laser irradiation. For postanesthetic recovery, rats received 5 mg/kg of ketoprofen for analgesia immediately before emergence and were monitored throughout recovery for gross behavioral deficits.

### Transdermal Laser Irradiation

For spinal cord irradiation, rats were positioned beneath two custom optical fibers that each delivered a rectangular beam (1 cm × 1 mm). The fibers were coupled to a Q-switched Nd:YAG laser (Tempest, New Wave Inc.) emitting 1064 nm laser pulses with a 10 Hz repetition rate and 5–7 ns pulse duration. The beams were oriented parallel along their longitudinal axes and angled such that their rectagular outputs converged at a focal depth of 1 cm. In air, the fibers delivered a fluence of approximately 70 mJ/cm^2^ at the spot of convergence. The combined fluence was below the ANSI maximum permissible exposure for skin at this wavelength (100 mJ/cm^2^).^24^ Each animal received 2400 pulses (4 min total). Animals undergoing laser treatment followed the postanesthetic recovery procedure described above.

### Ultrasound and Photoacoustic Imaging

For US/PA imaging, subjects were euthanized 24 h post laser irradiation. The L1, T13, and T12 laminae were removed to create an imaging window over the lumbar enlargement. Imaging was performed on the Vevo 2100 Imaging Platform with integrated Vevo LAZR pulsed laser (FUJIFILM VisualSonics, Inc.) using a LZ-550 linear array probe with center frequency of 40 MHz. For imaging, the transducer was gel-coupled to the exposed spinal cord. The system’s integrated Nd:YAG laser was operated at 1064 nm with a 20 Hz repetition rate and 5–7 ns pulse duration, delivering a fluence of 24 mJ/cm^2^. Images were acquired in coronal orientations. Sagittal images were generated through a multi-step process involving motorized stage acquisition, volumetric reconstruction, and orthogonal extraction. Specifically, a motorized stage was utilized to capture sequential coronal frames across the length of the cord; these frames were subsequently reconstructed into a 3D volume using VevoLab software (FUJIFILM VisualSonics, Inc.), from which the longitudinal sagittal planes were extracted.

### Histology and Confocal Microscopy

For histology and confocal microscopy, subjects were euthanized 4 h post laser irradiation. Spinal cords were excised from the L1 to T12 spinal levels and immediately snap-frozen in OCT compound (Tissue-Tek) using a dry ice and ethanol slurry. Immediate freezing was performed to minimize loss of delivered FITC-dextran that occurs with transcardial perfusion, chemical fixation, and cryoprotection.^25^ Frozen cords were sectioned on a cryostat (CM1860, Leica Biosystems) at 50 μm thickness in the rostral-to-caudal orientation. Sections were mounted with Fluoromount-G containing DAPI (SouthernBiotech) and imaged at 10× magnification using a Zeiss confocal laser scanning microscope 900 (Zeiss Group). DAPI was imaged using 405 nm excitation with emission collected from 400–510 nm, and FITC was imaged using 488 nm excitation with emission collected from 510–700 nm. Single-plane images were collected at a pixel size of 0.329 μm, with laser power and digital gain held constant across all sections to ensure uniform image acquisition.

### Ex Vivo Fluorescence Imaging

For ex vivo fluorescence imaging, subjects were euthanized twenty-4 h post laser irradiation. The L1, T13, and T12 laminae were removed to create an imaging window over the lumbar enlargement as done in the photoacoustic imaging experiments. Then, a segment of the spine containing exposed spinal cord was extracted. Imaging was perfomed using an IVIS Spectrum CT (PerkinElmer) in fluorescence imaging mode with Living Image software (PerkinElmer). FITC-dextran was detected using 465 nm excitation and 520 nm emission filters. All spinal cords were imaged simultaneously to enable direct quantitative comparison. Fluorescence and photograph images were processed using FIJI software.^26^ For quantitative analysis of FITC-dextran, nontissue background signal was first thresholded out using the built-in Huang’s method within FIJI. The mean signal of the saline-injected rat was then calculated and subtracted from every pixel. At this point, negative values were discarded to remove background autofluorescence signal originating from spinal cord tissue. Therefore, the remaining signal is generated by FITC-dextran. This processed image is shown in Figure 4B. Finally, the mean pixel signal for each spinal cord was computed and reported in Radiant Efficiency Units.

## Results and Discussion

### PFCnD Design and Characterization

We synthesized PFCnDs with a lipid monolayer shell, a perfluorohexane core, and an encapsulated NIR-absorbing dye. Perfluorohexane (boiling point: 56 °C) was selected to ensure that droplets vaporize under nanosecond laser excitation and reliably recondense at physiological temperature (37 °C), enabling the repeated cavitation cycles required for barrier permeabilization.^20^ We used lipids as the shell material to support a stable submicron-size, maintain structural integrity over repeated cavitation cycles, and minimize optical droplet vaporization (ODV) thresholds.^27,28^

Dynamic light scattering analysis showed that the synthesized PFCnDs exhibited Z-average hydrodynamic diameters of 281.8 nm (polydispersity index, PDI 0.10) at 25 °C and 297.8 nm (PDI 0.12) at 37 °C (Figure 1A). The visible–NIR absorption spectrum displayed a peak near 1064 nm, corresponding to the encapsulated Epolight 3072 dye and confirming optical tunability (Figure 1B). The 1064 nm absorption peaks enable both laser activation and high photoacoustic imaging contrast of the nanodroplets at the same wavelength. Nanoparticle tracking analysis of the synthesized nanodroplet solution measured a stock concentration of 3 × 10^11^ particles/mL.

**Figure 1:**
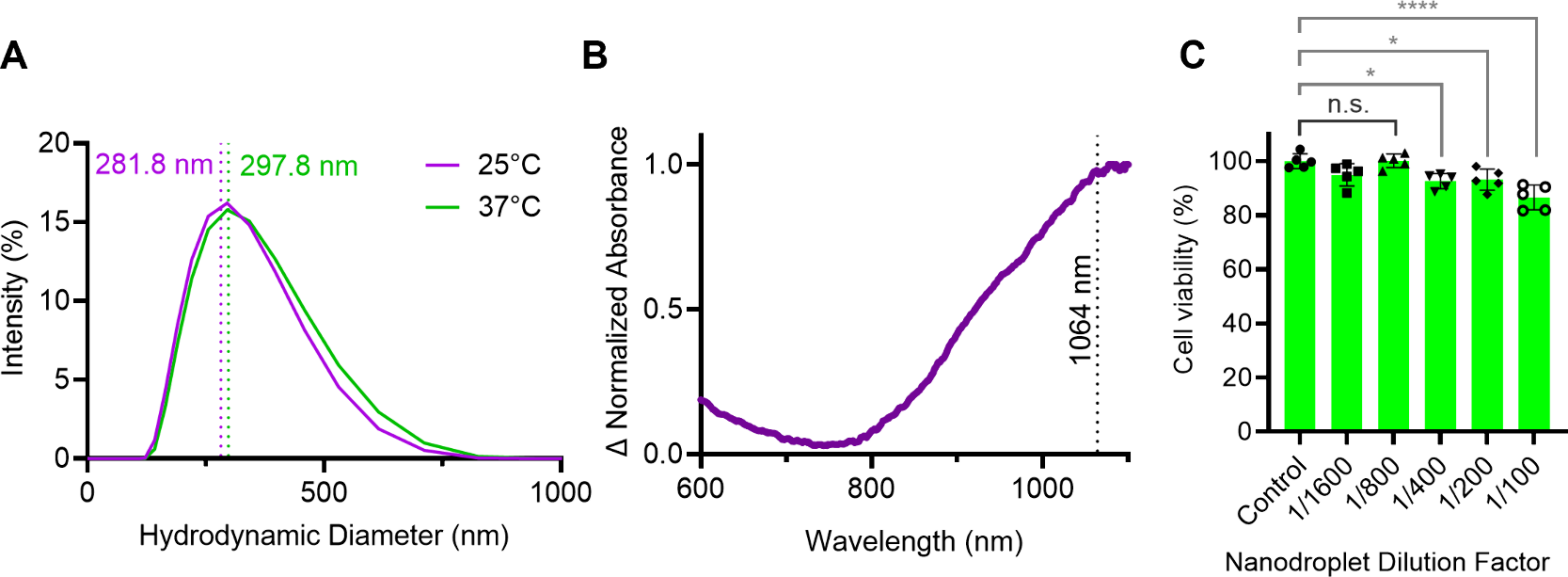
PFCnDs are optimized for controlled intrathecal spinal delivery. (A) Hydrodynamic diameter measured by DLS were 281.8 nm (PDI 0.10) at 25 °C and 297.8 nm (PDI 0.12) at 37 °C. (B) Differential visible–NIR absorbance spectrum of dye-loaded nanodroplets after subtraction of unloaded nanodroplet background, showing peak absorption near 1064 nm. (C) HEK293 viability after 24 h exposure to increasing nanodroplet concentrations (*n* = 5). No significant cytotoxicity was observed at dilutions of 1/800 or 1/1600 (*P* = 0.12 and >0.9999).

We evaluated cytotoxicity in HEK293 cells following 24 h exposure to PFCnDs at concentrations spanning the anticipated theraputic range (Figure 1C). One-way ANOVA indicated a significant overall effect of nanodroplet concentration on viability (F­(5,24) = 10.34, *P* < 0.05, n = 5 per group). However, Dunnett’s post hoc analysis revealed no significant difference between untreated controls and groups exposed to 1/1600 or 1/800 dilutions (*P* > 0.9999 and P = 0.12, respectively), indicating that PFCnDs are well-tolerated at concentrations used for subsequent in vivo studies.

### Laser Irradiation Produces Spatially Selective Nanodroplet Delivery

To assess whether laser-activated PFCnDs enable spatially controlled delivery, we used photoacoustic imaging to visualize the accumulation of PFCnDs in spinal cord tissue after intrathecal injection and targeted transdermal laser irradiation. Live rats received (1) an intrathecal injection of synthesized nanodroplets and (2) transdermal laser irradiation over the T13 vertebral region 30 min later. T13 was selected because it overlies a portion of the lumbar enlargement and is accessible for both irradiation and subsequent imaging.^29^ Then, 24 h postirradiation, rats were euthanized, and laminae were removed to expose the lumbar enlargement for ex vivo US/PA imaging (Figure 2).

**Figure 2:**
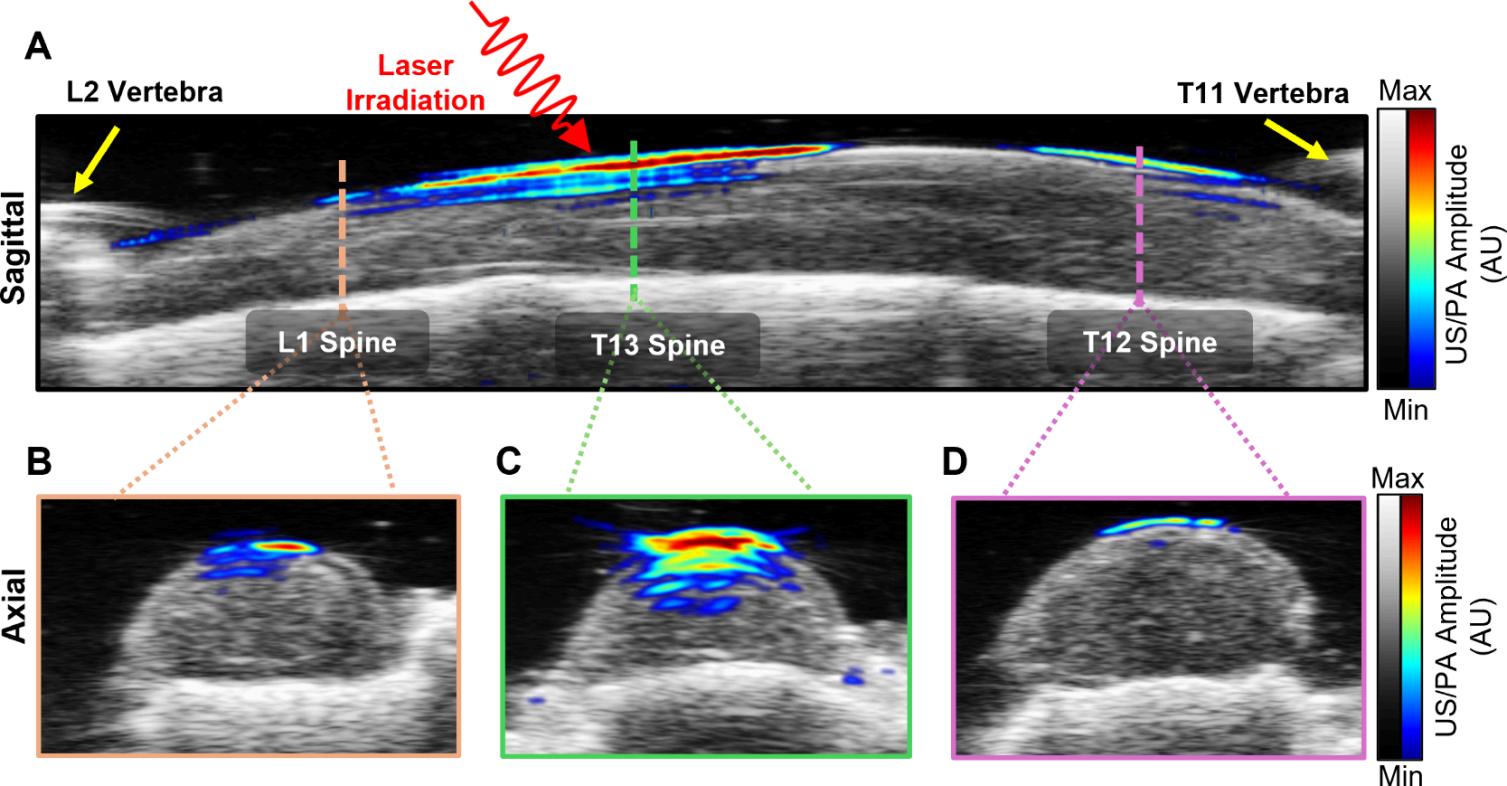
Intrathecal injection of nanodroplets followed by laser irradiation enables spinal delivery with spatial specificity. (A) Sagittal grayscale US and color PA (1064 nm excitation) image of a rat spinal cord after intrathecal nanodroplet injection and localized laser irradiation. Vertebral levels are indicated by yellow arrows to define the extent of preimaging laminectomy. (B–D) Axial US/PA images acquired at positions marked in (A). Note that except for the irradiation site (A, B, D), the PA signal is primarily confined to the dorsal subarachnoid space, whereas at the irradiation site (A, C) the PA signal extends into spinal cord parenchyma, demonstrating localized, laser-mediated intrathecal delivery.

Sagittal US/PA imaging revealed distinct patterns of nanodroplet distribution along the spinal cord (Figure 2A). At the site of laser irradiation near the L1–T13 interspace, strong PA signal extended into the spinal cord parenchyma (Figure 2A, C). Because photoacoustic amplitude increases linearly with the concentration of photoabsorbers, the observed increase in PA signal intensity indicates a localized increase in the concentration of delivered nanodroplets. In contrast, along nonirradiated segments of the cord, PA signal remained concentrated at the dorsal surface (Figure 2A, B, D), consistent with the presence of uncleared nanodroplets within the subarachnoid space. The confinement of intraparenchymal PA signal to the irradiated region demonstrates that spatial selectivity of delivery can be achieved and controlled by laser targeting.

These results also demonstrate the advantage of PFCnDs as a photoacoustic contrast agent. Here, this contrast enabled whole-field spine assessment of delivery. The PA signal generated by dye-loaded nanodroplets upon laser excitation provides a built-in reporter of nanodroplet distribution and tissue penetration.^23^ This dual functionality, controlled delivery combined with imaging feedback, could support image-guided treatment protocols in future clinical applications, allowing confirmation of successful delivery during the procedure.

### Intraparenchymal Delivery Requires Both Nanodroplets and Laser Activation

To isolate the contributions of nanodroplets and laser irradiation, we evaluated four experimental conditions: (1) intrathecal saline injection without laser, (2) saline with laser, (3) nanodroplets without laser, and (4) nanodroplets with laser (n = 3 per group). Transdermal laser irradiation was directed over the L1 vertebral level, which contains a portion of the lumbar enlargement.^29^ We measured nanodroplet distribution in spinal cord axial cross sections with US/PA imaging (Figure 3A).

**Figure 3:**
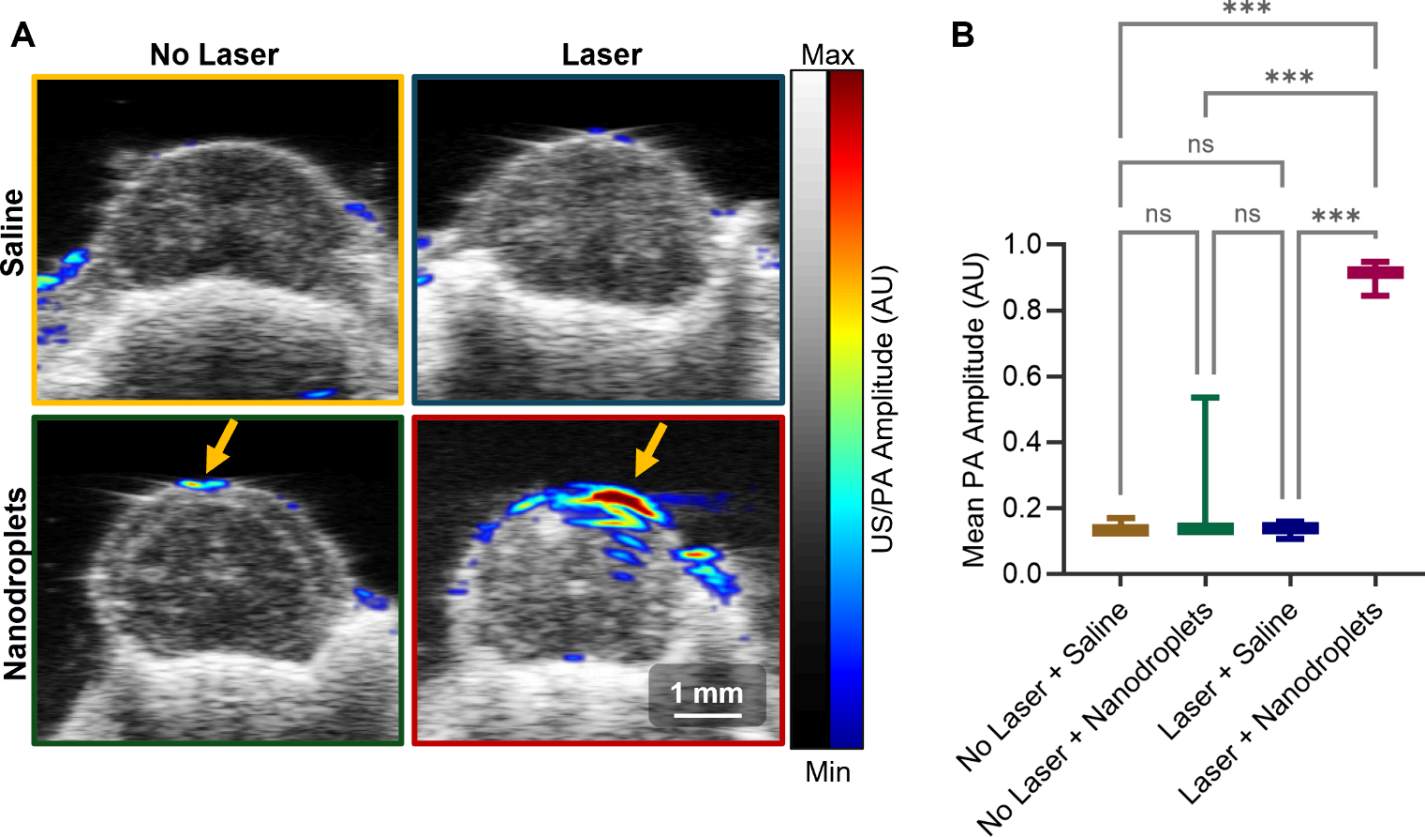
Intraparenchymal spinal cord delivery requires nanodroplets activation by laser irradiation. (A) Cross sections of the L1 spinal cord showing nanodroplet distribution across four treatment groups: saline or nanodroplet intrathecal injection, with or without transdermal laser irradiation. Grayscale US intensity shows spinal anatomy; color PA signal (1064 nm excitation) overlay indicates nanodroplet localization. Yellow arrow points to nanodroplet signal. Only the Laser + Nanodroplets group exhibited detectable PA signal within the spinal cord. (B) Comparison of mean PA signal amplitudes within the spinal cord (*n* = 3 per group). Laser + Nanodroplets produced significantly higher PA signal compared to all other groups (***p < 0.001, one-way ANOVA with Tukey’s post hoc test). There are no significant differences among the other groups. Center lines indicate medians, and whiskers extend to the full data range (min to max). When the minimum equals the median, the lower whisker is not visible.

Saline-injected controls displayed no detectable PA signal regardless of laser exposure, confirming that the observed contrast originated specifically from dye-loaded nanodroplets. In rats receiving nanodroplet injections without laser activation, weak PA signal was confined to the subarachnoid space, indicating that nanodroplets alone do not penetrate the spinal parenchyma. In contrast, the combination of nanodroplet injection with laser irradiation produced strong PA signal extending into the spinal cord tissue.

Quantitative analysis of mean PA amplitude within the segmented spinal cord region revealed that the nanodroplet-plus-laser group exhibited approximately 7-fold higher signal compared to all other groups (Figure 3B; ***P < 0.001, one-way ANOVA with Tukey’s post hoc test). No significant differences were observed among the three control groups. These results demonstrate that intraparenchymal delivery requires the interaction between laser irradiation and intrathecally administered nanodroplets.

### Laser-Activated Nanodroplets Enable Tunable Delivery of Large Macromolecules

To determine whether laser-activated PFCnDs could facilitate delivery of therapeutically relevant macromolecules, we used confocal microscopy to observe penetration of a 500 kDa FITC-labeled dextran intrathecally coadministered with PFCnDs, followed by laser irradiation as described above. The FITC-dextran-500 kDa reporter was selected for its hydrodynamic diameter of approximately 30 nm,^30^ falling within the size range of therapeutically relevant macromolecules including viral vectors. Transdermal laser irradiation was directed at the left dorsal cord while leaving the right dorsal spinal cord unexposed to allow for comparison. Four hours postirradiation, spinal cords were excised, sectioned, and imaged.

In the nonirradiated section, FITC-dextran remained confined to the subarachnoid space, forming a fluorescent outline around the cord perimeter (Figure 4A, left). Occasional narrow tracks of FITC signal penetrated the superficial parenchyma, consistent with passive accumulation in Virchow-Robin spaces. This pattern indicates that macromolecules near 30 nm in diameter can enter Virchow-Robin spaces but do not access deeper spinal cord parenchyma without induced barrier permeabilization.

**Figure 4:**
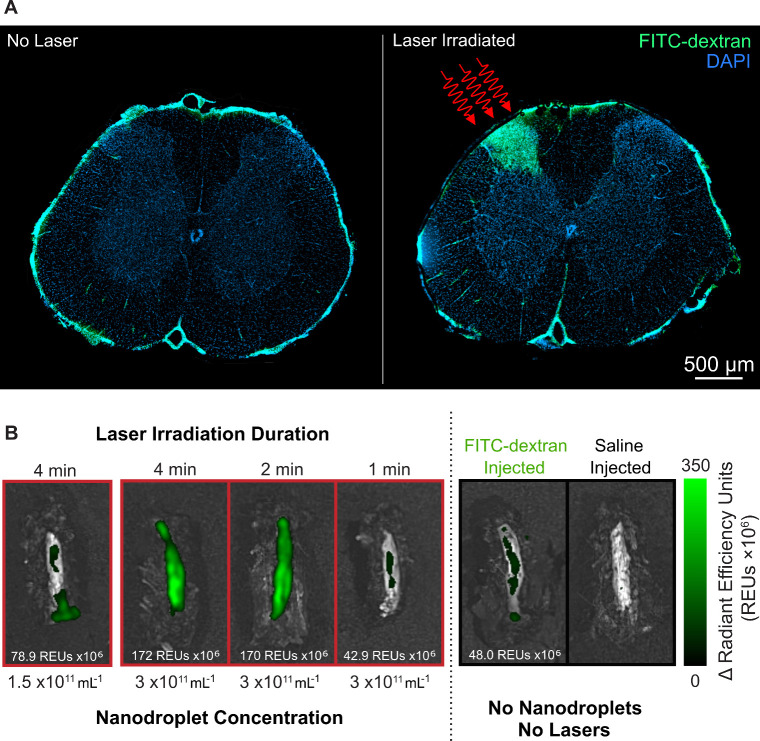
Laser-activated nanodroplets enable tunable intrathecal spinal delivery of large macromolecules. (A) Representative transverse spinal cord sections from the same rat following intrathecal injection of nanodroplets coadministered with FITC-dextran-500 kDa (∼30 nm diameter, green). Sections were stained with DAPI (blue). (A: left) The spinal cord section not exposed to laser irradiation showing FITC signal mostly confined to the subarachnoid space, with small tracks of FITC entering the cord, consistent with Virchow-Robin space accumulation. (A: right) The spinal cord section exposed to transdermal laser irradiation showing deep intraparenchymal delivery of FITC-dextran at the site of laser irradiation (red arrows). (B) Ex vivo fluorescence imaging of rat spinal cord tissue (L1–T13 vertebral segments, laminectomy-exposed) following intrathecal injection, demonstrating dose-dependent intraparenchymal delivery. All pixels are background-subtracted against the mean signal from the saline-injected control. Mean net radiant efficiency (REUs × 10^6^) is annotated on each image. (B: left) Cords receiving FITC-dextran and nanodroplets at varying concentrations (mL^–1^) and irradiation durations (min). Signal increases with irradiation duration at the higher nanodroplet concentration and scales with nanodroplet dose at 4 min. (B: right) Controls receiving FITC-dextran or saline without nanodroplets or laser irradiation.

By contrast, irradiated sections exhibited robust FITC signal within the left dorsal horn, extending approximately 1 mm into the parenchyma on the laser-exposed side (Figure 4A, right). The contralateral (nonirradiated) side of the same sections showed minimal intraparenchymal signal, further confirming the spatial specificity of laser-mediated delivery.

To assess the dose-dependence of delivery, we performed ex vivo fluorescence imaging of spinal cord tissue using an IVIS imaging system across independent rats receiving varied nanodroplet concentrations and irradiation durations (Figure 4B). At the higher nanodroplet concentration (3 × 10^11^ particles/mL), fluorescent signal increased with irradiation duration. The signal observed at 1 min irradiation was comparable to that of animals receiving FITC-dextran without nanodroplets or laser irradiation. This is consistent with residual FITC-dextran confined to the subarachnoid space that had not been cleared at the time of imaging, as observed in the nonirradiated confocal sections (Figure 4A). This indicates that 1 min of irradiation is insufficient to produce meaningful barrier permeabilization above passive accumulation. At 2 and 4 min, signal plateaued, suggesting that delivery approaches saturation at the higher concentration beyond 2 min of irradiation. At 4 min of irradiation, reducing the nanodroplet concentration by a factor of 2 (3 × 10^11^ to 1.5 × 10^11^ particles/mL) yielded approximately half the signal, demonstrating that nanodroplet dose independently contributes to the amount of delivered material. Together, these results indicate that intraparenchymal delivery of 500 kDa dextran is tunable across both nanodroplet concentration and irradiation duration, with a minimum threshold of irradiation duration required to produce delivery above passive accumulation.

Three complementary imaging modalities were used to characterize macromolecular delivery at different scales. Confocal fluorescence microscopy provided high-resolution visualization of FITC-dextran penetration depth in spinal cord sections (Figure 4A). Photoacoustic imaging of PFCnDs enabled quantitative assessment of delivery localization across spinal segments (e.g., L1 versus L2) due to its larger field of view, though at coarser resolution (Figures 2 and 3). Ex vivo fluorescence imaging permitted quantification of total FITC-dextran delivery across treatment conditions (Figure 4B).

### Mechanism of Large Molecule Delivery

We propose that the mechanism driving the delivery of large molecules into the spinal cord tissue is permeabilization of the glia limitans and associated barriers at the CSF-spinal cord interface. In this model, once the barrier is opened, large molecules diffuse into the spinal cord tissue down their concentration gradient. Therapeutics near 30 nm in diameter should be able to penetrate throughout spinal cord tissue as studies have measured CNS extracellular space to have an effective pore size of about 38–64 nm in width.^31^ The precise cellular barriers being modulated, whether the glia limitans, pia mater, or a combination, remain to be determined in future mechanistic studies.

Our finding that neither nanodroplets alone nor laser irradiation alone produced intraparenchymal delivery supports a cavitation-dependent mechanism (Figure 3A). The 7-fold increase in PA signal within the spinal parenchyma observed only in the combined treatment group (Figure 3B) indicates that laser activation of nanodroplets is required to overcome the barriers restricting macromolecular entry. Delivery appears to occur through vaporization-induced cavitation: upon absorption of pulsed NIR laser energy, PFCnDs undergo rapid liquid-to-gas phase transition, generating localized mechanical forces that transiently permeabilize nearby cellular barriers.^20,21^ The high-boiling-point perfluorohexane core enables recondensation at physiological temperature, allowing repeated cavitation cycles during the irradiation period.

The dose-dependence of delivery further supports this mechanism (Figure 4B). Intraparenchymal fluorescent signal increased with both nanodroplet concentration and irradiation duration, consistent with greater cavitation-induced mechanical stress on the barrier arising from more cavitation agents and more cavitation events, respectively. However, delivery plateaued between 2 and 4 min of irradiation. At least two explanations may account for this nonlinear relationship. First, the barrier may reach a maximum degree of permeabilization beyond which additional cavitation events produce no further macromolecular entry. Second, nanodroplets may undergo structural degradation during sustained laser pulsing, reducing the number of active cavitation agents available by the 2 min time point and limiting further delivery. The abundance of coadministered FITC-dextran makes cargo availability an unlikely limiting factor. Distinguishing between these mechanisms will require future studies using real time PA monitoring to characterize in vivo nanodroplet integrity over the irradiation period.

### Therapeutic Implications

The successful delivery of 500 kDa FITC-dextran (∼30 nm hydrodynamic diameter) suggests applicability to therapeutics of similar or smaller sizes (Figure 4). Adeno-associated viruses, the most widely used vectors for CNS gene therapy, have diameters of approximately 20 nm.^32^ The approximately 1 mm penetration depth achieved in the dorsal horn (Figure 4) is therapeutically relevant, as many spinal cord pathologies, including motor neuron diseases, neuropathic pain conditions, and traumatic injuries, involve neuronal populations within this range of the cord surface.

The spatial selectivity demonstrated here (Figure 2) could enable targeted treatment of specific spinal segments while sparing others. This may be particularly valuable for conditions with focal pathology, such as localized spinal cord injuries or segmental motor neuron degeneration, where widespread vector distribution is neither necessary nor desirable given the immunogenic potential of viral vectors.^3^

The approach described here is compatible with clinically accessible procedures. Intrathecal injections via lumbar puncture are routinely performed for diagnostic and therapeutic purposes, and Q-switched Nd:YAG lasers are widely available in medical settings. The laser fluences used in this study were within ANSI Z136.1 maximum permissible exposure limits for skin,^24^ supporting the feasibility of transdermal activation.

### Limitations and Future Directions

While we demonstrated delivery of a size-matched fluorescent reporter, we did not assess delivery of functional gene therapy vectors or measure transgene expression. Future studies will evaluate AAV delivery and quantify transduction efficiency in target cell populations.

Our safety assessment was limited to in vitro cytotoxicity (Figure 1C); comprehensive histopathological evaluation of treated spinal cord tissue, including assessment of inflammation, hemorrhage, and tissue architecture, will be essential for translational development.

The temporal dynamics of barrier opening were not characterized. Understanding the duration of the permeabilization window and the timeline of barrier recovery will be important for optimizing treatment protocols.

Off-target biodistribution of intrathecally administered therapeutics has been characterized in previous studies.^33^ However, translational development of our approach will require confirmation that the intervention does not adversely alter systemic biodistribution of specific therapeutics in off-target peripheral organs.

This study focused on delivery of ∼30 nm macromolecules. Future studies should investigate the size threshold and dynamics of macromolecules that could be delivered with this technique. However, the effective pore sizes of the extracellular space (38–64 nm) would limit particles much larger than the 30 nm dextran used here.^31^ Translation to larger animal models and ultimately humans will require consideration of increased tissue depth. The optical energy required to activate nanodroplets at greater depths will necessitate alternative delivery strategies, such as integrating fiber-optics with intrathecal catheters, which are already established for use in clinical settings for pain management.^5^

## Conclusion

We have shown that laser-activated perfluorocarbon nanodroplets enable spatially selective delivery of macromolecules into the spinal cord parenchyma after intrathecal administration without surgical exposure. By combining intrathecal administration with transdermal NIR laser irradiation, we achieved localized delivery without laminectomy or direct surgical access to the spinal cord. Laminectomy was only needed in this study for visualization and verification. Using a ∼30 nm fluorescent reporter, we showed delivery of macromolecular cargo to depths of ∼1 mm in the dorsal horn. These results establish a foundation for future development of targeted spinal gene therapies, RNA medicines, and other biologics currently limited by intrathecal transport barriers.

## Glossary

BBB: blood–brain barrier
CNS: central nervous system
CSF: cerebrospinal fluid
FUS: focused US
MBs: microbubbles
NIR: near-Infrared
ODV: optical droplet vaporization
PA: photoacoustic
PFCnDs: perfluorocarbon nanodroplets
US: ultrasound
